# Deep multimodal fusion of image and non-image data in disease diagnosis and prognosis: a review

**DOI:** 10.1088/2516-1091/acc2fe

**Published:** 2023-04-11

**Authors:** Can Cui, Haichun Yang, Yaohong Wang, Shilin Zhao, Zuhayr Asad, Lori A Coburn, Keith T Wilson, Bennett A Landman, Yuankai Huo

**Affiliations:** 1 Department of Computer Science, Vanderbilt University, Nashville, TN 37235, United States of America; 2 Department of Pathology, Microbiology and Immunology, Vanderbilt University Medical Center, Nashville, TN 37215, United States of America; 3 Department of Biostatistics, Vanderbilt University Medical Center, Nashville, TN 37215, United States of America; 4 Division of Gastroenterology Hepatology, and Nutrition, Department of Medicine, Vanderbilt University Medical Center, Nashville, TN 37232, United States of America; 5 Department of Electrical and Computer Engineering, Vanderbilt University, Nashville, TN 37235, United States of America; 6 Veterans Affairs Tennessee Valley Healthcare System, Nashville, TN 37212, United States of America

**Keywords:** multimodal learning, fusion method, medical data, diagnosis and prognosis

## Abstract

The rapid development of diagnostic technologies in healthcare is leading to higher requirements for physicians to handle and integrate the heterogeneous, yet complementary data that are produced during routine practice. For instance, the personalized diagnosis and treatment planning for a single cancer patient relies on various images (e.g. radiology, pathology and camera images) and non-image data (e.g. clinical data and genomic data). However, such decision-making procedures can be subjective, qualitative, and have large inter-subject variabilities. With the recent advances in multimodal deep learning technologies, an increasingly large number of efforts have been devoted to a key question: how do we extract and aggregate multimodal information to ultimately provide more objective, quantitative computer-aided clinical decision making? This paper reviews the recent studies on dealing with such a question. Briefly, this review will include the (a) overview of current multimodal learning workflows, (b) summarization of multimodal fusion methods, (c) discussion of the performance, (d) applications in disease diagnosis and prognosis, and (e) challenges and future directions.

## 1. Introduction

Routine clinical visits of a single patient might produce digital data in multiple modalities, including image data (i.e. pathology images, radiology images, and camera images) and non-image data (i.e. lab test results and clinical data). The heterogeneous data would provide different views of the same patient to better support various clinical decisions (e.g. disease diagnosis and prognosis [1-3]). However, such decision-making procedures can be subjective, qualitative, and exhibit large inter-subject variabilities [4, 5]. With the rapid development of artificial intelligence technologies, an increasingly large amount of deep learning-based solutions has been developed for multimodal learning in medical applications. Deep learning includes high-level abstraction of complex phenomenon within high-dimensional data, which tends to benefit multimodal fusion in extracting and modeling the complex relationships of different modalities and outcomes [6, 7].

**Figure 1.**
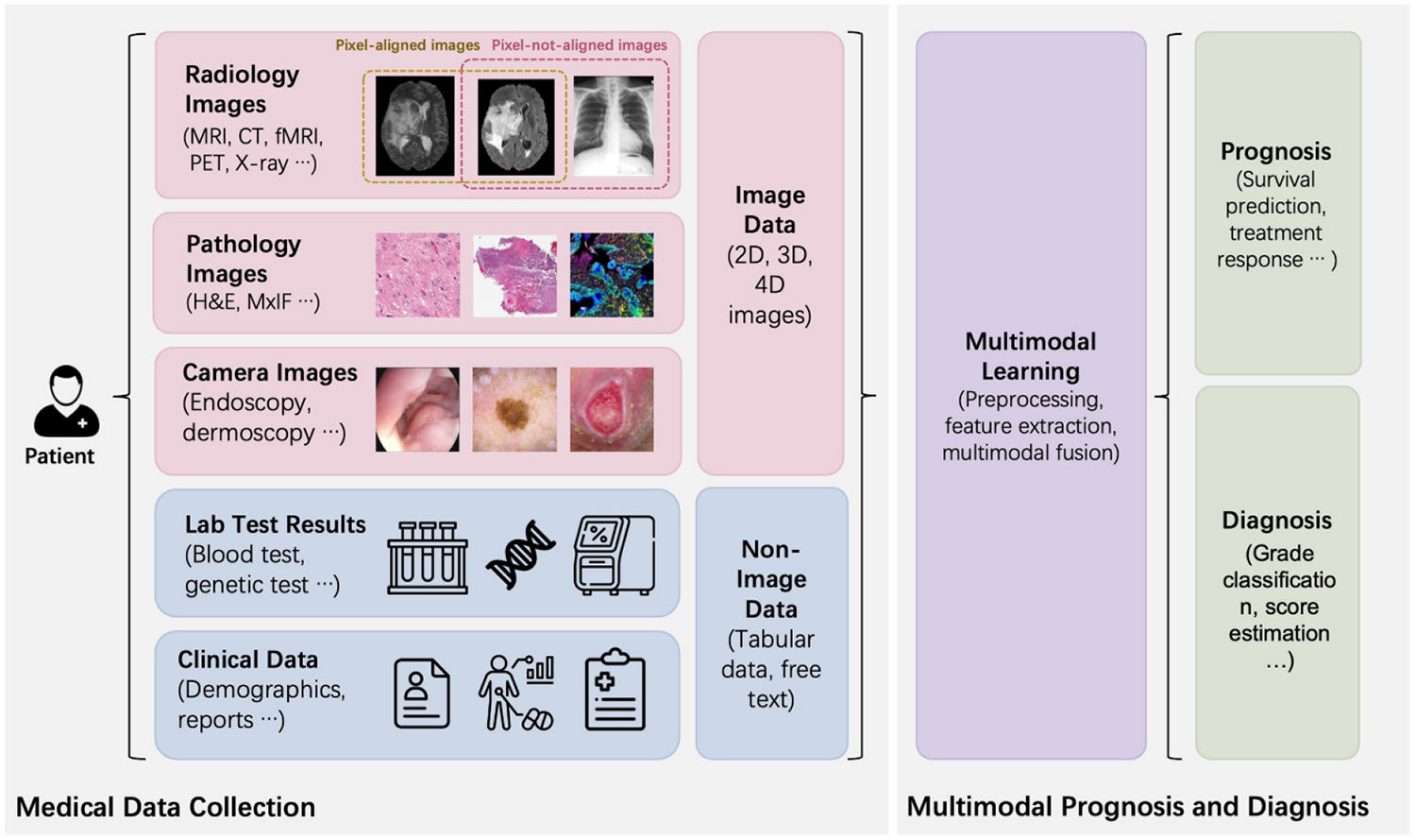
The scope of this review is presented. multimodal data containing image data (e.g. radiology images and pathology images) and non-image data (e.g. genomic data and clinical data) are fused through multimodal learning methods for diagnosis and prognosis of diseases.

Many works have achieved great success in using a single modality to make a diagnosis or prognosis with deep learning methods [8-10]. However, fusing the multimodal data effectively is not a trivial task in method design because different clinical modalities may contain different information (complementary information of a subject) and have different data formats. Figure 1 summarizes the scope of this review: that the multimodal data (image and non-image data) from the same patient are utilized for diagnosis or prognosis of diseases. The image data can be categorized as radiology image data, pathology image data, and camera image data. Such imaging data can be further classified as pixel-aligned data (can be spatially registered and overlayed) and pixel-not-aligned data (the pixels in different images do not have spatial correspondence), which might even have different dimensionalities (e.g. 2D, 3D, and 4D). The non-image data can be categorized as lab test results such as structured genomic sequences and blood test results, and clinical data including tabular data of demographic features, or free text in the lab test reports. The heterogeneity of such image and non-image data leads to critical challenges in performing multimodal learning, a family of algorithms in machine learning. For example, 2D pathology images provide micro-level morphology for a tumor while the 3D radiology images such as computed tomography (CT)/magnetic resonance imaging (MRI) offer macro-level and spatial information of the same tumor. The clinical data and lab test results indicate the molecular, biological, and chemical characteristics, while the structured DNA and mRNA sequences are also involved in clinical decision making. Moreover, image data are typically larger and denser (e.g. millions of pixels), while the non-image data are more sparse with a lower dimensionality. Herein, the heterogeneous formats (e.g. different dimensions, image, free text, and tabular data) require different preprocessing and feature extraction methods, and different types of information require fusion methods that are able to capture the shared and complementary information effectively for rendering better diagnosis and prognosis.

Several surveys have been published for medical multimodal fusion [11-15]. Boehm *et al* [11] reviewed the applications, challenges, and future direction of multimodal fusion in oncology. Huang *et al* [12] and Stahlschmidt *et al* [15] categorized the fusion methods by the stages of fusion. Schneider *et al* [13] and Lu *et al* [14] divided the multimodal learning studies by downstream tasks and modalities. In this survey, we not only adhere to the widely recognized stages of fusion categorization, but also review multimodal fusion techniques from a new perspective of categorizing the feature-level fusion methods into operation-based, subspace-based, tensor-based, and graph-based fusion methods. We hope the summary of the fusion techniques can foster new methods in medical multimodal learning.

In this survey, we collected and reviewed 34 related works published within the last 5 years. All of them used deep learning methods to fuse image and non-image medical data for prognosis, diagnosis, or treatment prediction. This survey is organized in the following structure: section 2 provides an overview of multimodal learning for medical diagnosis and prognosis; section 3 briefly introduces the data preprocessing and feature extraction for different unimodalities, which is the prerequisite for multimodal fusion; section 4 summarizes categorized multimodal fusion methods, and their motivation, performance, and limitations are discussed; section 5 provides a comprehensive discussion and future directions; and section 6 is a conclusion.

## 2. Overview

### 2.1. Study selection

This survey only includes published studies (with peer-review) that fuse both image and non-image data to make a disease diagnosis or prognosis in the past five years. All of them used feature-level deep learning-based fusion methods for multimodal data. A total of 34 studies that satisfied these criteria are reviewed in this survey.

### 2.2. Workflow

**Figure 2.**
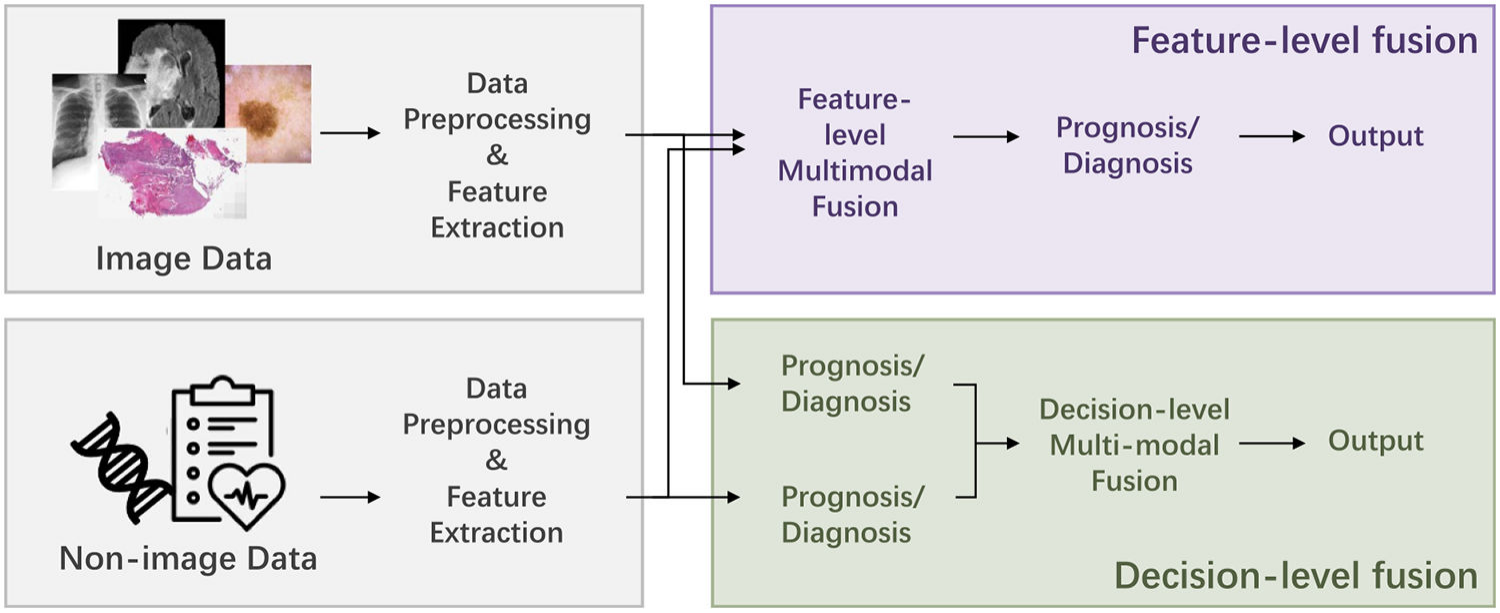
The overview of the multimodal learning workflow is presented. Due to the heterogeneity of different modalities, separate preprocessing methods and feature extraction methods are used for each modality. For feature-level fusion, the extracted features from unimodals are fused. Note that the feature extraction methods can be omitted, because some data can be fused directly (such as the tabular clinical features). As for decision-level fusion, the different modalities are fused in the probability or final predication level. Because feature-level fusion contains more variants of fusion strategies, we mainly focus on reviewing the feature-level fusion methods, but also compares with the decision-level fusion.

A generalized workflow of collected studies is shown in figure 2. Typically, data preprocessing, unimodal feature extraction, multimodal fusion, and predictor sections are included in the workflow. Due to the heterogeneity of image and non-image modalities, it is unusual to fuse the original data directly. Different modalities always have separate methods of data preprocessing and feature extraction. For multimodal learning, fusion is a crucial step, following the unimodal data preprocessing and feature extraction steps that are the prerequisites. Sections 3 and 4 will introduce and discuss the unimodal feature preparation and multimodal fusion separately.

Based on the stage of multimodal fusion, the fusion strategies can be divided into feature-level fusion and decision-level fusion [12, 15]. Feature-level fusion contains early fusion and intermediate fusion. For decision-level fusion, which is also referred to as late fusion, the prediction results of unimodal models (e.g. probability logits or categorical results from unimodal paths in classification tasks) are fused for multimodal prediction by majority vote, weighted sum, or averaging, etc. The fusion operation is relatively simple and there is no need to retrain the unimodal models at the fusion stage. As for feature-level fusion, either the extracted high-dimensional features or the original structured data can be used as the inputs. Compared with decision-level fusion, feature-level fusion has the advantage of incorporating the complementary and correlated relationships of the low-level and high-level features of different modalities [12, 16], which leads to more variants of fusion techniques. This survey mainly focuses on categorizing the methods of feature-level fusion, but also compares with the decision-level fusion.

### 2.3. Diagnosis and prognosis tasks and evaluation metrics

In this survey, multimodal fusion is applied to the disease diagnosis and prognosis. The disease diagnosis tasks include classifications such as disease severity, benign or malignant tumors, and regression of clinical scores. Prognosis tasks include survival prediction and treatment response prediction.

After obtaining the multimodal representations, multi-layer perceptrons (MLP) were used by most of the reviewed studies to generate the prognosis or diagnosis results. The specific tasks of diagnosis and prognosis can be categorized into regression or classification tasks based on discrete or continuous outputs. To supervise the modal training, the cross-entropy loss is usually used for classification tasks, while the mean square error (MSE) is a popular choice for regression tasks. To evaluate the results, the area under the curve (AUC) of receiver operating characteristics (ROC), mean average precision (mAP), accuracy, F1-score, sensitivity, and specificity metrics are commonly used for classification, while the MSE is typically used for regression. However, although the survival prediction is treated as a time regression task or a classification task of long-term/short-term survival, the Cox proportional hazards loss function [17] is popular in survival prediction tasks. To evaluate the survival prediction models, the concordance index (c-index) is widely used to measure the concordance between the predicted survival risk and real survival time.

## 3. Unimodal data preprocessing and feature extraction

Due to multimodal heterogeneity, separate preprocessing and feature extraction methods/networks are required for different modalities to prepare unimodal features for fusion. As shown in table 1, our reviewed studies contain image modalities such as pathology images (H&E), radiology images (CT, MRI, x-ray, functional MRI (fMRI)), and camera images (clinical images, macroscopic images, dermatoscopic images); and non-image modalities such as lab test results (genomic sequences) and clinical features (free-text reports and demographic data). In this section, we briefly introduce these data modalities and summarize the corresponding data preprocessing and feature extraction methods.

### 3.1. Image data

#### 3.1.1. Pathology images

Pathology images analyze cells and tissues at a microscopic level, which is recognized as the ‘gold standard’ for cancer diagnosis [19]. The 2D Hematoxylin-Eosin (H&E) stained pathology images with 3 channels is a widely used one. Yet, the whole slide image (WSI) of pathology images usually cannot be processed directly because of its gigantic size. So, the smaller patches are always cropped from the informative regions of interests (ROIs) of WSIs to fit the computation memory. To define the ROIs, some reviewed studies used the diagnostic ROIs manually annotated by experts [20, 21] or predicted by the pre-trained segmentation models [21, 48], while some reviewed studies instead selected ROIs from the foreground [18, 44] or dense region [25, 47] based on pixel intensity. Also, the color space can be converted from RGB to HSV for H&E images for higher intensity contrast [18, 44], and always standardized [26, 67] or calibrated to standard images [17, 21] for data consistency. To generate features from cropped image patches, 2D conventional neural networks (CNNs) are popular options for learning-based features. Moreover, finetuning models pre-trained by natural images (e.g. ImageNet [96]) is usually preferred especially for small datasets [26, 44, 48, 68], though there is a concern for the large gap between natural images and pathology images [27]. Except for the learning-based methods, some other works [28, 29] used conventional feature extraction methods (e.g. CellProfiler [30]) to extract statistical and structured features about shapes, texture, intensity, etc. Meanwhile, multiscale is a common mechanism to extract complementary and shared information from different level of pathology images [20, 27, 31, 48]. As for the supervision, if only the WSI-level label is provided for patches (e.g. the patches from ROIs extracted based on intensity), multi-instance learning (MIL) is always applied to aggregate the information of patches in bags for supervision [32, 33].

#### 3.1.2. Radiology images

Radiology imaging supports medical decisions by providing visible image contrasts inside the human body with radiant energy, including MRI, CT, positron emission tomography (PET), fMRI and x-ray, etc. To embed the intensity standardized 2D or 3D radiology images into feature representations with learning-based encoders [16, 24, 34-36, 87] or conventional radiomics methods [24, 34, 35] or both [34, 35], skull-stripping [38], affine registration [38], foreground extraction [39], lesion segmentation [20, 34, 35, 38] were used correspondingly in some reviewed works to define the ROIs at first. And then, the images were resized or cropped to a smaller size for feature extraction. In order to further reduce the image dimension to fit computation memory but keep essential information, some works used 2D maximum intensity projection [16], and some works used the representative slices with maximum tumor diameters [20, 35] to convert the 3D volume to 2D. The fMRI is another radiology modality used in some reviewed studies that investigated autism spectrum disorder (ASD) and Alzheimer’s disease (AD) [40, 41]. The images of brains were divided into multiple regions by the template. Then, the Pearson correlation coefficient between two brain regions was calculated to form the functional connectivity matrix, and the matrix was finally vectorized for classification.

#### 3.1.3. Camera images

In addition to pathology and radiology images, some other kinds of medical images captured by optical color cameras are categorized as camera images; examples of these camera images include dermoscopic and clinical images for skin lesions [42, 45], endoscopic images to examine the interior of a hollow organ or cavity of the body [49], and the funduscopic images photographing the rear of eyes [50]. Different from pathology images, camera images can be taken directly while the sectioned and stained sample slides are not required. Also, most of the camera images are three-channel color images but in a smaller size than pathology images. 2D pre-trained CNN networks [42] or pre-trained transformer models [51] are usually applied to the whole images or detected lesions.

### 3.2. Non-image data

The non-image modalities contain lab test results and clinical features. Laboratory tests check a sample of blood, urine, or body tissues, access the cognition and psychological status of patients, and analyze genomic sequences, etc. Clinical features include demographic information and clinical reports. These modalities are also essential to diagnosis and prognosis in clinical practice. The non-image data on the reviewed works can be briefly divided into structured data and free-text data for different preprocessing and feature extraction methods.

#### 3.2.1. Structured data

Most of the clinical data and lab test results in the reviewed works are structured data and can be converted to feature vectors easily. In preprocessing, categorical clinical features were usually converted through one-hot encoding, while the numerical features were standardized [16, 34, 35]. Specially, Cai *et al* [51] used soft one-hot encoding by setting the elements that were as 0 in the standard one-hot encoding as 0.1 to make contributions to the propagation. As the genomic data are in high dimension, some feature selection methods such as the highest variance were used to extract the most informative features [44, 47, 67]. The missing value is a common problem for some structured data. The ones with a high missing rate were usually discarded directly, while the other missing data were imputed with the average value, mode value, or values of similar samples selected by K-nearest neighbors [16, 24], and some works added missing status as features [52, 68].

#### 3.2.2. Free-text data

Clinical reports capture clinicians’ impressions of diseases in the form of unstructured text. In order to deal with the free text data and extract informative features from the free-text, natural language processing techniques are implemented. For example, Chauhan *et al* [53] prepared the tokenization of the text extracted by ScispaCy [54]. Then, the BERT [55] model initialized by weights pre-trained on the scientific text [56] was used to embed the tokenization. Furthermore, the language model trained by medical data such as ClinicalBERT [57] was tried in the work [58] for text embeddings and compared its performance with BERT in multimodal prediction.

After using the above modal-specific preprocessing and feature extraction methods, the unimodal representations could be converted to feature maps or feature vectors. For feature vectors, in order to learn more expressive features with expected dimensions, EI-Sappagh *et al* [24] used principal component analysis (PCA), to reduce the dimension of radiomics features. Parisot *et al* [40] explored different feature reduction methods such as recursive feature elimination, PCA, and the autoencoder to reduce the feature dimension of the vectorized functional connectivity matrix. In contrast, Yan *et al* [27] used the denoising autoencoder to enlarge the dimension of low-dimensional clinical features to avoid being overwhelmed in feature-level fusion by high-dimensional image features. For similar purpose, Yoo *et al* [38] replicated and scaled the clinical features, while Cui *et al* [68] deconvoluted the feature vectors to the same size as feature maps of image modalities. In terms of aggregating multiple feature vectors, El-Sappagh *et al* [24] used bidirectional long short-term memory (biLSTM) models to handle the vectorized time-series features, and Lu *et al* [18] applied attention-based MIL to accumulate the feature vectors in bags to bag-level representations. As for the image feature maps learned by CNNs, these feature maps could be used for fusion directly in order to keep the spatial information [59, 68], vectorized with pooling layers [16, 34, 35], or split by pixel/voxels as the tokens to feed transformers [58].

Unimodal feature extraction can be either unsupervised or supervised. Note that if the unimodal features are trained prior to fusion, it is possible to train the unimodal model with the maximum number of available samples of each modality for better unimodal model performance and hopefully better unimodal features to benfit the multimodal performance [35, 67, 84]. Regarding the relationship of unimodal feature extraction with the fusion, the unimodal feature extraction section can be independent to the fusion section [35, 87], which is known as early-level fusion, or can be trained from scratch or finetuned with the fusion section end-to-end [20, 31] as the intermediate-level fusion.

## 4. Multimodal fusion methods

**Figure 3.**
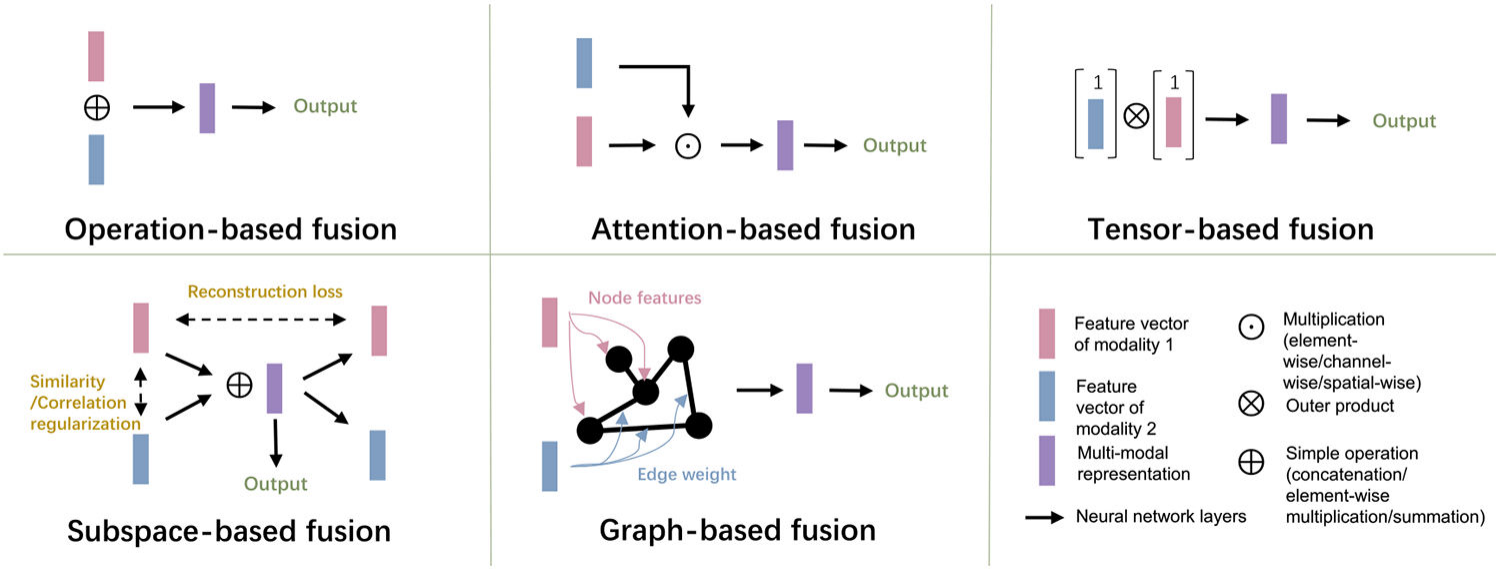
Representative structures of different feature-level multimodal fusion methods.

Fusing the heterogeneous information from multimodal data to effectively boost prediction performance is a key pursuit and challenge in multimodal learning [97]. Based on the type of inputs for multimodal fusion, the fusion strategies can be divided into feature-level fusion and decision-level fusion [11]. Decision-level fusion integrates the probability or categorical predictions from unimodal models using simple operations such as averaging, weighted vote, majority vote, or a meta classifier with trainable layers for unimodal probability [16, 45, 60, 61, 87, 98], to make a final multimodal prediction. For the decision-level fusion, the prediction of unimodality can be learned separately and be independent to the fusion stage. It can fuse any combination of multimodalities without further adjustment in the testing phase. So, it may be preferable for flexibility and simplicity, and it can tolerate the missing modality situation. Sometimes the decision-level fusion achieved better performance than the feature-level fusion. For example, Wang *et al* [60] implemented a learnable weighted sum mechanism based on unimodal uncertainty to fuse the prediction of different modalities, which outperformed the intermediate feature-level fusion. Huang *et al* [87] showed that decision-level fusion also outperformed feature-level fusion in their experiments of pulmonary embolism detection. However, the decision-level fusion may lack the interaction of the features. For the modalities with dependent or correlated features, feature-level fusion might be more preferable. Some other works [16, 61] also showed that their proposed feature-level integration performed better than decision-level fusion. On the other hand, feature-level fusion fuses the original data or extracted features of heterogeneous multimodals into a compact and informative multimodal hidden representation to make a final prediction. Compared with decision-level fusion, more variants of feature-level fusion methods have been proposed to capture the complicated relationship of features from different modalities. This survey reviews these methods and categorizes them into operation-based, subspace-based, attention-based, tensor-based, and graph-based methods. The representative structures of these fusion methods are displayed in figure 3, and the fusion methods of reviewed studies are summarized in table 1.

### 4.1. Operation-based fusion methods

To combine different feature vectors, the common practice is to perform simple operations of concatenation, element-wise summation, and element-wise multiplication. These practices are parameter-free and flexible to use, but the element-wise summation and multiplication methods always require the feature vectors of different modalities to be converted into the same shape. Many early works used one of the simple operations to show that multimodal learning models outperforms unimodal models [16, 18, 21, 24, 38, 42, 45, 87]. Although the operation-based fusion methods are simple and effective, they might not exploit the complex correlation between heterogeneous modalities. Also, the long feature vectors generated by the concatenation may lead to overfitting when the amount of training data is not sufficient [62, 63]. More recently, Holste *et al* [16] compared these three operation-based methods in the task of using clinical data and MRI images for breast cancer classification. The low-dimensional non-image features were processed by fully connected layers (FCN) to the same dimension of image features before fusion. The results showed that the three operations performed comparably (*p*-value > 0.05), while the element-wise summation and multiplication methods required less trainable parameters in the following FCN. After comparing the learned non-image features by FCN and the original non-image features, the former ones achieved superior performance. Meanwhile, the concatenation of the feature vectors outperformed the concatenation of logits from the unimodal data. Yan *et al* [27] investigated the influence of the dimension of unimodal features on the unimodal performance using concatenation fusion. They hypothesized that the high-dimensional vectors of image data would overwhelm the low-dimension clinical data. To keep the rich information of the high-dimensional features for a sufficient fusion, they used the denoising autoencoder to increase the dimension of clinical features. Zhou *et al* [84] proposed a three-stage unimodal feature learning and multimodal feature concatenation pipeline, where every two modalities were fused at the second stage and all three modalities were fused at the third stage, in order to use the maximum number of available samples when some modalities were missing.

### 4.2. Subspace-based fusion methods

The subspace methods aim to learn an informative common subspace of multimodality. A popular strategy is to enhance the correlation or similarity of features from different modalities. Yao *et al* [28] proposed a DeepCorrSurv model and evaluated the survival prediction task. Inspired by the conventional canonical correlation analysis (CCA) method [99], they proposed an additional CCA-based loss for the supervised FCN network to learn the more correlated feature space of features from two modalities. The proposed methods outperformed the conventional CCA methods by learning the non-linear features and the supervised correlated space. Zhou *et al* [39] designed two similarity losses to enforce the learning of modality-shared information. Specifically, a cosine similarity loss was used to supervise the features learned from these two modalities, and a loss of hetero-center distance was designed to penalize the distance between the center of clinical features and CT features belonging to each class. In their experiments, the accuracy dropped from 96.36 to 93.18 without these similarity losses. Li *et al* [48] used the average of L1-norm and L2-norm loss to improve the similarity of the learned unimodal features from pathology images and genes before concatenating them as a multimodal representation. The learned similar features can then be fused by concatenation as the multimodal representation. Another study [47] fused the feature vectors from 4 modalities with the subspace idea in the diagnosis task of 20 cancer types. Inspired by the SimSiam network [64], they forced the feature vectors from the same subject to be similar by a margin-based hinge-loss. Briefly, cosine similarity scores between the unimodal features from the same patient were maximized, whereas the ones from different patients were minimized. The feature similarities of different patients were only penalized within a margin of the feature similarity. Such a regularity enforced similar feature representation from the same patient, while avoiding mode collapse.

Another strategy in the subspace-based fusion method is to learn a complete representation subspace with the encoder-decoder structures. Ghosal *et al* [65] decoded the mean vectors of multimodal features and used the reconstruction loss to force the mean vectors to contain the complete information of different views. The mean vectors with additional decoder and reconstruction loss achieved superior classification accuracy as compared with the counterparts without such loss functions. Similarly, Cui *et al* [35] also used the autoencoder backbone to learn the complete representation, but some modalities were randomly dropout and reconstructed by the mean vector generated from the available modalities to improve prediction accuracy and be more robust to the testing data with missing modalities.

### 4.3. Attention-based fusion methods

Attention-based methods computed and incorporated the importance scores (attention scores) of multimodality features when performing aggregation. This progress simulated routine clinical practice. For example, the information from clinical reports of a patient may inform the clinicians to pay more attention to a certain region in an MRI image. Duanmu *et al* [59] built an FCN path for non-image data along with a CNN path for image data. The learned feature vectors from the FCN path were employed as the channel-wise attention for the CNN path at the corresponding layers. The low-level and high-level features of different modalities can be fused correspondingly, which achieved a better prediction accuracy than simple concatenation. Schulz *et al* [20] concatenated the learned feature vectors from three modalities by an attention layer, which weighted the modal importance for the downstream task. Chen *et al* [26] calculated the co-attention weight to generate the genomic-guided WSI embeddings. Similarly, Lu *et al* [69] proposed a symmetric cross attention to fuse the genomic data and pathology image embeddings of glioma tumors for multitask learning, while Cai *et al* [51] proposed an asymmetrical multi-head cross attention to fuse the camera images and metadata for skin classification. Li *et al* [31] aggregated multiscale pathology images and clinical features to predict the lymph node metastasis (LNM) of breast cancer. In their proposed method, the clinical features and patient-level image representations via mean pooling were concatenated to form the global multimodal representations, which were to guide the attention-based MIL of image patches and recalibrate the clinical features. The experiments showed that the proposed attention-based methods outperformed both the gating-based attention used by Chen *et al* [67] and a bag-concept layer concatenation [70]. Guan *et al* [36] applied the self-attention mechanism [100] in their concatenated multimodal feature maps. They tiled and transformed the clinical feature vectors to the same shape of the image feature matrix to keep the spatial information in an image feature map. Their performance surpassed both the concatenation and another subspace method using a similarity loss [53]. In addition to the MLP and CNN, the attention mechanism was also applied to the graph model for multimodal learning in the medical domain. Cui *et al* [68] built a graph where each node was composed of image features and clinical features with category-wise attention. The influence weights of neighboring nodes were learned by the convolution graph attention network (con-GAT) and novel correlation-based graph attention network (cor-GAT). The attention value was used to update the node features for the final prediction. More recently, the transformer models were widely used in multimodal learning [71], and they were adapted to medical field. Jacenkow *et al* [72] exploited the unimodally pre-trained transformer-based language model BERT [55] and finetuned it after adding the image tokens for multimodal learning. Li *et al* [58] used the radiology images and radiology free-text reports to finetune the visual-text transformer models pre-trained by general image-language pairs not specific for medical domains. Meanwhile, different visual-text backbone, unimodal pre-trained models and training strategy were compared in their work.

The above attention-based fusion methods rescaled features through complementary information from another modality, while Pölsterl *et al* [52] proposed a dynamic affine transform module that shifted the feature map. The proposed modules dynamically produced scale factor and offset conditional on both image and clinical data. In such a design, the affine transform was added ahead of the convolutional layer in the last residual block to rescale and shift the image feature maps. As a result, the high-level image features can interact with the compacted clinical features, which outperformed the simple concatenation and channel-wise attention-based methods [59].

### 4.4. Tensor-based fusion methods

The tensor-based fusion methods conducted outer products across multimodality feature vectors to form a higher order co-occurrence matrix. The high-order interactions tend to provide more predictive information beyond what those features can provide individually. For example, blood pressure rising is common when a person is doing high-pressure work, but it is dangerous if there are also symptoms of myocardial infarction and hyperlipidemia [39]. Chen *et al* [67] proposed pathomic fusion to make prognosis and diagnosis utilizing pathology images, cell graphs, and genomic data. They used the tensor fusion network with a Kronecker product [73] to combine the unimodal, bimodal, and trimodal features. To further control the expressiveness of each modality, a gated-attention layer [74] was added. Wang *et al* [29] not only used the outer product for inter-modal feature interactions, but also for intra-modal feature interactions. It surpassed the performance of the CCA-based method known as DeepCorrSurv [28]. More recently, Braman *et al* [34] followed the work of pathomic fusion [67] and extended it from three modalities to four modalities. Also, an additional orthogonal loss was added to force the learned features of different modalities to be orthogonal to each other, which helped to improve feature diversity and reduce feature redundancy. They showed that their methods outperformed the simple concatenation and the original Kronecker product.

### 4.5. Graph-based fusion method

A graph is a non-grid structure to catch the interactions between individual elements represented as nodes. For disease diagnosis and prognosis, nodes can represent the patients, while the graph edges contain the associations between these patients. Different from CNN-based representation, the constructed population graph updates the features for each patient by aggregating the features from the neighboring patients with similar features. To utilize complementary information in the non-imaging features, Parisot *et al* [40] proposed to build the graph with both image and non-image features to predict ASD and AD. The nodes of the graph were composed of image features extracted from fMRI images, while the edges of the graph were determined by the pairwise similarities of image (fMRI) and non-image features (age, gender, site, and gene data) between different patients. Specifically, the adjacency matrix was defined by the correlation distance between the subject’s fMRI features multipled with the similarity measure of non-image features. Their experiment showed that the proposed graph convolutional network (GCN) model outperformed MLP of multimodal concatenation. Following this study, Cao *et al* [41] built graphs similarly but proposed to use the edge dropout and DeepGCN structure with residual connection instead of the original GCN for deeper networks and thus avoid overfitting, which achieved better results.

## 5. Discussion and future work

In the above sections, we reviewed recent studies using deep learning-based methods to fuse image and non-image modalities for disease prognosis and diagnosis. The feature-level fusion methods were categorized into operation-based, subspace-based, attention-based, tensor-based, and graph-based methods. The operation-based methods are intuitive and effective, but they might yield inferior performance when learning from complicated interactions of different modalities’ features. However, such approaches (e.g. concatenation) are still used to benchmark new fusion methods. Tensor-based methods represent a more explicit manner of fusing multimodal features, yet with an increased risk of overfitting. Attention-based methods not only fuse the multimodal features but compute the importance of inter- and intra-modal features. Subspace-based methods tend to learn a common space for different modalities. The current graph-based methods employ graph representation to aggregate the features by incorporating prior knowledge in building the graph structure. Note that these fusion methods are not exclusive to each other, since some studies combined multiple kinds of fusion methods to optimize the prediction results. Compared with decision-level fusion for the decision-level fusion, feature-level fusion may gain benefits from the interaction between multimodal features, while the decision-level fusion is more flexible for the combination of multimodalities and thus robust to modality missing problems.

Although different fusion methods have different characteristics, how to select the optimal fusion strategy is still an open question in practice. There is no clue that a fusion method always performs the best. Currently, it is difficult to compare the performance of different fusion methods directly, since different studies were typically done on different datasets with different settings. Moreover, most of the prior studies did not use multiple datasets or external testing sets for evaluation. Therefore, more fair and comparative studies and benchmark datasets should be encouraged for multimodal learning in the medical field. Furthermore, the optimal fusion method might be task/data dependent. For example, the decision-level fusion might be more suitable for multimodality with less correlation. However, the theoretical analysis and evaluation metrics are not extensively researched. Some studies show that the fusion at different layers or levels can significantly influence the results [16, 59, 84, 87]. The neural architecture search provides an option to automatically optimize the network structure. It has been applied in the image and non-image fusion in other fields [101-103], but it is under explored in multimodal medical applications.

The reviewed studies showed that the performance of multimodal models typically surpassed the unimodal counterparts in the downstream tasks such as disease diagnosis or prognosis. On the other hand, some studies also mentioned that the models fused more modalities may not always perform better than those with fewer modalities. In other words, the fusion of some modalities may have no influence or negative influence on multimodal models [18, 20, 34, 40, 41]. It might be because the additional information introduces bias for some tasks. For example, Lu *et al* [18] used the data of both primary and metastatic tumors for training to increase the top-k accuracy of the classification of metastatic tumors effectively. However, the accuracy decreased by 4.6% when biopsy site, a clinical feature, was added. Parisot *et al* [40] and Cao *et al* [41] demonstrated that the fusion of redundant information or data with noise (e.g. age, full intelligence quotient) led to defining inaccurate neighborhood systems of the population graph and further decreased the model performance. Meanwhile, additional modalities increase the network complexity with more trainable parameters, which may increase the training difficulties and the risk of overfitting. Braman *et al* [34] used outer products to fuse unimodal features. However, the outer products with three modalities yielded an inferior performance compared with the pairwise fusion and even unimodal models. Thus, although multimodal learning tends to benefit model performance, modality selection should consider the model capacity, data quality, specific tasks, etc. This is still an interesting problem worth more exploration.

A concern in this field is data availability. Although deep learning is powerful in extracting a pattern from complex data, it requires a large amount of training data to fit a reasonable model. However, data scarcity is always a challenge in the healthcare area, the situation of the multimodal data is only worse. Over 50% of the reviewed studies used multimodal datasets containing less than 1,000 patients. To improve the model performance and robustness with limited data, the pre-trained networks (e.g. image encoder Transformers and CNN networks pre-trained by natural images [96], text encoder BERT [55] and ClinicalBERT [57], and multimodal encoder VisualBERT [91], LXMERT [92] and UNITER [93] pre-trained by natural image-visual pairs) were widely used by many studies instead of training from scratch with small datasets. Meanwhile, several studies [18, 44, 45, 69] deployed multi-task learning and showed improvements. Through sharing representations between related tasks, models generalized better on the original task. Also, many studies applied feature reduction and data augmentation techniques to avoid overfitting. To enlarge the paired dataset scale for multimodal fusion, combining multi-site data is a straightforward method, but the computational data harmonization is worth to consider to eliminate the non-biological variances of multi-site data for a general and robust model [104]. Also. unsupervised methods [105] and semi-supervised methods [106] which have gained a great success in unimodal learning can also be applied to utilize the multimodal data without labels. Meanwhile, transfer learning with larger datasets shared related knowledge can benefit the multimodal learning. For example, Sharifi-Noghabi *et al* [107] used multimodal pan-drug data in training to enlarge the multimodal dataset and achieve better performance of the response prediction in a drug-specific task than the model trained by drug-specific data only. Similarly, Cheerla *et al* [47] noticed that the survival prediction of single cancers was improved by using all cancer data instead of using the single cancer data for training. Data missing is another problem of data availability. Complete datasets with all modalities available for every patient are not always guaranteed in routine practice. In the reviewed papers, random modality dropout [35, 47], multi-stage training [34, 35, 84], partial network training [65, 84], data imputation [24], and autoencoders with reconstruction loss [35, 65] have been implemented to handle the missing data in multimodal learning. However, the comparison of these methods and the influence of missing data in training and testing phases were not thoroughly investigated. Utilizing limited data effectively and efficiently is a practical but essential problem. This is a fast-growing direction attracting more and more attention.

Unimodal feature extraction is an essential prerequisite for fusion, especially for multimodal heterogeneity. Proper preprocessing and feature extraction methods/networks are inevitable for the following fusion procedures. Both the standard feature extraction methods and learning-based feature extraction methods are commonly seen in deep fusion works. According to some reviewed works, different feature extraction methods can influence the fusion results significantly. For example, Cai *et al* [51] observed that the ViT-based image encoder led to better fusion results than CNN-based encodes, and the fusion model using clinical features with soft one-hot encoding also outperformed hard encoding and word2vec. Li *et al* [58] compared the contribution of different pre-trained language models to multimodal fusion. The results showed that although the language model CliniclBERT pretrained by medical data outperformed the BERT model in unimodal prediction, it does not fit the pre-trained weights in the fusion stage and performed slightly worse in multimodal prediction. To boost the fusion prediction, unimodal preprocessing and feature extraction should be carefully designed and evaluated. For better unimodal representation, different strategies can be considered. For example, the segmentation results tend to benefit the diagnostic feature extraction and ease the unimodal learning by providing regions of interests [34, 35, 39, 108]. The combination of conventional radiomics features and learning-based features achieved better performance [34, 39]. And the unimodal encoder trained by more data is more capable for representation generalization [35, 84, 105, 106]. Large unimodal datasets are always easier to obtain than multimodal dataset. The leading techniques can be adapted to the unimodal feature extraction to boost the multimodal performance.

Explainability is another challenge in multimodal diagnosis and prognosis. Lack of transparency is identified as one of the main barriers to deploying deep learning methods in clinical practice. An explainable model not only provides a trustworthy result but also helps the discovery of new biomarkers. In the reviewed papers, some explanation methods were used to show feature contributions to results. For image data, heatmaps generated with the class activation maps algorithm (CAM) were used to visualize the activated region of images that were most relevant to the models’ outputs [20, 53, 67]. Li *et al* [31] displayed the attention scores of patches to visualize the importance of every patch to the MIL of a WSI. The activated image region was compared with prior knowledge to see whether the models focused on the diagnostic characteristics of images. For non-image features, Holste *et al* [16] used a permutation-based measure, Ghosal *et al* [65] used learnable dropout layers, while Zhou *et al* [39] and Chen *et al* [67] implemented a gradient-based saliency method to get the score of feature importance. Especially, explainability in multimodal learning helps explain and visualize the interaction between different modalities, EI-Sappagh *et al* [24], Cheerla etand Gevaert [47] and Braman *et al* [34] displayed the importance of modalities with the performance of multimodal models trained by different combination of modalities. Chen *et al* [26] visualized the correlation of gene data and pathology image regions to reflect the known genotype-phenotype relationships of cancers. Although the usefulness of these explanations is still waiting to be validated in clinical practice, the development of more advanced meta-explanation through multimodal information fusion can be a promising topic for future study as Yang *et al* [109] mentioned in their medical explainable AI review.

## 6. Conclusion

This paper has surveyed the recent works of deep multimodal fusion methods using the image and non-image data in medical diagnosis, prognosis, and treatment prediction. The multimodal framework, multimodal medical data, and corresponding feature extraction were introduced, and the deep fusion methods were categorized and reviewed. From the prior works, multimodal data typically yielded superior performance as compared with the unimodal data. Integrating multimodal data with appropriate fusion methods could further improve the performance. On the other hand, there are still open questions to achieve a more generalizable and explainable model with limited and incomplete multimodal medical data. In the future, multimodal learning is expected to play an increasingly important role in precision medicine as a fully quantitative and trustworthy clinical decision support methodology.

## Figures and Tables

**Table 1.** An overview of image and non-image modalities, number of subjects, tasks multimodal fusion methods and performance comparison of reviewed studies (the performance are reported by reviewed studies).

	Study	Modalities	Subjects	Tasks	Fusion strategy	Fusion details	Performance comparison (uni-/multimodal)	Performance comparison (different fusion methods)
1	Holste *et al* [16]	MRI images, clinical features	17,046 samples of 5,248 patients	Classification of breast cancer	Operation	Element-wise multiplication/elementwise summation/concatenation of learned unimodal features or direct features.	[AUC] Images: 0.860, clinical features: 0.806, all: 0.903 (*P*-value < 0.05)	[AUC] learned feature concatenation: 0.903, sum: 0.902, multiplication: 0.896; probability fusion: 0.888 (*p*-value >0.05)
2	Lu *et al* [18]	H&E images, clinical features	1) 32,537 samples of 29,107 patients from CPTAC [22] and TCGA [23]. (Public) 2) 19162 samples of 19162 patients from an in-house dataset. 3) External testing set: 682 patients.	Classification of primary and metastatic tumors, and origin sites.	Operation	Concatenation of clinical features and the learned pathology image feature.	[Top-1 accuracy] Image: about 0.740, image + sex: about 0.808, image + sex + site: about 0.762 (Metastatic tumors)	—
3	EI-Sappagh *et al* [24]	MRI and PET images, neuropsychology data, cognitive scores, assessment data	1,536 patients from ADNI [37]. (Public)	Classification of AD and prodromal status. Regression of 4 cognitive scores.	Operation	Concatenation of learned static features and learned time-series unimodal features from five stacked CNN-biLSTM.	[Accuracy] Five modalities: 92.62, four modalities: 90.45, three modalities: 89.40, two modalities: 89.09. (Regression performance is consistent with the classification)	—
4	Yan *et al* [27]	Pathology images, clinical features	3,764 samples of 153 patients. (Public)	Classification of breast cancer.	Operation	Concatenation of increased-dimensional clinical features and multi-scale image features.	[Accuracy] Image + clinical features: 87.9, clinical features: 78.5, images: 83.6	—
5	Mobadersany *et al* [21]	H&E images, genomic data	1,061 samples of 769 patients from the TCGA-GBM and TCGA-LGG [23]. (Public)	Survival prediction of glioma tumors	Operation	Concatenation of genomic biomarkers and learned pathology image features.	[C-Index] Image: 0.745, gene: 0.746, images + gene: 0.774, (*P*-value < 0.05)	—
6	Yap *et al* [42]	Macroscopic images, dermatoscopic images, clinical features	2,917 samples from ISIC [43]. (Public)	Classification of skin lesion.	Operation	Concatenation of clinical features and learned image features.	[AUC] Dsc + macro + clinical: 0.888, dsc + macro: 0.888, macro: 0.854, dsc: 0.871	—
7	Silva *et al* [44]	Pathology images, mRNA, miRNA, DNA, copy number variation (CNV), clinical features	11,081 patients of 33 cancer types from TCGA [23]. (Public)	Pancancer survival prediction.	Operation Attention	Attention weighted element-wise summation of unimodal features.	[C-Index] Clinical: 0.742, mRNA: 0.763, miRNA: 0.717, DNA: 0.761, CNV: 0.640, pathology: 0.562, clinical + mRNA + DNAm: 0.779, all six modalities: 0.768	—
8	Kawahara *et al* [45]	Clinical images, dermoscopic images, clinical features	1,011 samples. (Public)	Classification of skin lesion.	Operation	Concatenation of learned unimodal features.	[Accuracy] Clinical images + clinical features: 65.3, dermoscopic images + clinical features: 72.9, all modalities: 73.7	—
9	Yoo *et al* [38]	MRI images, clinical features	140 patients	Classification of brain lesion conversion.	Operation	Concatenation of learned images features and the replicated and rescaled clinical features.	[AUC] Images: 71.8, images + clinical: 74.6	—
10	Yao *et al* [28]	Pathology images, genomic data	1) 106 patients from TCGA-LUSC. 2) 126 patients from the TCGA-GBM [23]. (Public)	Survival prediction of lung cancer and brain cancer.	Operation Subspace	Maximum correlated representation supervised by the CCA-based loss.	[C-index] Pathology images: 0.5540, molecular: 0.5989, images + molecular: 0.6287. (LUSC). Similar results on other two datasets.	[C-index] Proposed: 0.6287, SCCA [46]: 0.5518, DeepCorr + DeepSurv [17]: 0.5760 (LUSC). Similar results on other two datasets.
11	Cheerla *et al* [47]	Pathology images, genomic data, clinical features	11,160 patients from TCGA [23] (nearly 43% of patients miss modalities). (Public)	Survival prediction of 20 types of cancer.	Operation Subspace	The average of learned unimodal features, while a margin-based hinge-loss was used to regularize the similarity of learned unimodal features.	[C-index] Clinical + miRNA + mRNA + pathology: 0.78, clinical + miRNA: 0.78, clinical + mRNA: 0.60, clin + miRNA + mRNA:0.78, clinical + miRNA + pathology: 0.78	—
12	Li *et al* [48]	Pathology images genomic data	826 cases from the TCGA-BRCA [23]. (Public)	Survival prediction of breast cancer.	Operation Subspace	Concatenated the learned unimodal features regularized by a similarity loss.	[C-index] Images + gene: 0.7571, gene: 0.6912, image: 0.6781. (*p*-value < 0.05)	—
13	Zhou *et al* [39]	CT images, laboratory indicators, clinical features	733 patients	Classification of COVID-19 severity.	Operation Subspace	Concatenated the learned unimodal features regularized by a similarity loss.	[Accuracy] Clinical features: 90.45, CT + clinical features: 96.36	[Accuracy] Proposed: 96.36, proposed wo/similarity loss: 93.18
14	Ghosal *et al* [65]	Two fMRI paradigms images, genomic data (single nucleotide polymorphisms (SNP))	1) 210 patients from the LIBD institute. 2) External testing set: 97 patients from BARI institute.	Classification of neuropsychiatric disorders.	Operation Subspace	Mean vector of learned unimodal features, supervised by the reconstruction loss.	—	[AUC] Proposed: 0.68, encoder + dropout: 0.62, encoder only: 0.59 (LIBD). The external test set showed the same trend of results.
15	Cui *et al* [35]	H&E and MRI images, genomic data (DNA), demographic features	962 patients (170 with complete modalities) from TCGA-GBMLGG [23] and BraTs [66] (Public)	Survival prediction of glioma tumors.	Operation Subspace	Mean vector of learned unimodal features with modality dropout, supervised by the reconstruction loss.	[C-index] Pathology: 0.7319 radiology: 0.7062, DNA: 0.7174, demographics: 0.7050, all: 0.7857	[C-index] Proposed: 0.8053, pathomic fusion [67]: 0.7697, deep orthogonal [34]: 0.7624
16	Schulz *et al* [20]	CT, MRI and H&E images, genomic data	1)230 patients from the TCGA-KIRC [23]. (Public) 2) External testing set: 18 patients.	Survival prediction of clear-cell renal cell carcinoma.	Operation Attention	Concatenation of learned unimodal features with an attention layer.	[C-index] Radiology: 0.7074, pathology: 0.7424, rad + path: 0.7791. (*p*-value < 0.05). The external test set showed similar results	—
17	Cui *et al* [68]	CT images, clinical features	924 samples of 397 patients	Lymph node metastasis prediction of cell carcinoma.	Operation Attention	The concatenation of learned unimodal features with a category-wise contextual attention were used as the attributes of graph nodes.	[AUC] Images: 0.782, images + clinical: 0.823.	[AUC] Proposed: 0.823, logistic regression: 0.713, attention gated [74]: 0.6390, deep insight [75]: 0.739
18	Li *et al* [31]	H&E images, clinical features	3,990 cases	Lymph node metastasis prediction of breast cancer.	Operation Attention	Attention-based MIL for WSI-level representation, whose attention coefficients were learned from both modalities.	[AUC] Clinical: 0.8312, image: 0.7111, clinical and image: 0.8844	[AUC] Proposed: 0.8844, concatenation: 0.8420, gating attention [67]: 0.8570, M3DN [70]: 0.8117
19	Duanmu *et al* [59]	MRI images, genomic data, demographic features.	112 patients	Response prediction to neoadjuvant chemotherapy in breast cancer.	Operation Attention	The learned feature vector of non-image modality was multiplied in a channel-wise way with the image features at multiple layers.	[AUC] Image: 0.5758, image and non-image: 0.8035	[AUC] Proposed: 0.8035, concatenation: 0.5871
20	Guan *et al* [36]	CT images, clinical features	553 patients	Classification of esophageal fistula risk.	Operation Attention	Self-attention on the concatenation of learned unimodal features. Concatenation of all paths in the end.	[AUC] Images: 0.7341, clinical features [76]: 0.8196, images + clinical: 0.9119	[AUC] Proposed: 0.9119, Concate: 0.8953, Ye *et al* [77]: 0.7736, Chauhan *et al* [53]: 0.6885, Yap *et al* [42]:0.8123
21	Pölsterl *et al* [52]	MRI images, clinical features.	1,341 patients for diagnosis and 755 patients for prognosis. (Public)	Survival prediction and diagnosis of AD.	Operation Attention	Dynamic affine transform module.	[C-index] Images: 0.599, images + clinical: 0.748	[C-index] Proposed: 0.748, FiLM [78]: 0.7012, Duanmu *et al* [59]: 0.706, concatenation: 0.729
22	Wang *et al* [79]	X-ray images, free-text reports.	1) Chest x-ray 14 dataset [80]. 2) 900 samples from a hand-labeled dataset. 3) 3,643 samples from the OpenI [81]. (Partially public)	Classification of thorax disease.	Operation Attention	Multi-level attention for learned features of image and text.	[Weighted accuracy] Text reports: 0.978, images: 0.722, images + text reports: 0.922. (Chest X-rays14). Similar results on other two datasets.	—
23	Chen *et al* [67]	H&E images, genomic data (DNA and mRNA)	1) 1,505 samples of 769 patients from TCGA-GBM/LGG. 2) 1,251 samples of 417 patients from TCGA-KIRC [23]. (Public)	Survival prediction and grade classification of glioma tumors and renal cell carcinoma.	Operation Attention Tensor Fusion	Kronecker product of different modalities. And a gated-attention layer was used to regularize the unimportant features.	[C-index] Images (CNN): 0.792, images (GCN): 0.746, gene: 0.808,images + gene: 0.826. (GBM/LGG) Similar results on the other dataset.	[C-index]: Proposed: 0.826, Mobadersany *et al* [21]: 0.781. (*p*-value < 0.05) (GBM/LGG). Similar results on the other dataset.
24	Wang *et al* [29]	Pathology images, genomic data	345 patients from TCGA [23](Public)	Survival prediction of breast cancer.	Operation Tensor Fusion	Inter-modal features and intra-modal features produced by the bilinear layers.	[C-index] Gene: 0.695, images: 0.578, gene + images: 0.723	[C-index] Proposed: 0.723, LASSO-Cox 0.700, inter-modal features: 0.708, DeepCorrSurv [28] : 0.684, MDNNMD [82]: 0.704, concatenation: 0.703
25	Braman *et al* [34]	T1 and T2 MRI images, genomic data (DNA), clinical features	176 patients from TCGA-GBM/LGG [23] and BraTs [66]. (Public)	Survival prediction of brain glioma tumors.	Operation Attention Tensor Fusion	Extended the fusion method in [67] to four modalities and the orthogonal loss was added to encourage the learning of complementary unimodal features.	[C-index] Radiology: 0.718, pathology: 0.715, gene: 0.716, clinical: 0.702, path + clin: 0.690, all: 0.785	[C-index] Proposed: 0.785, pathomic fusion [67]: 0.775, concatenation: 0.76
26	Cao *et al* [41]	fMRI images, clinical features	871 patients from ABIDE [83]. (Public)	Classification of ASD and health controls.	Graph Operation	Nodes features were composed of image features, while the edge weights were calculated by images and non-image features.	[Accuracy] Sites + gender + age + FIQ: 0.7456, sites + age + FIQ: 0.7534, sites + age: 0.7520	[Accuracy] Proposed: 0.737, Parisot *et al* [40]: 0.704
27	Parisot *et al* [40]	fMRI images, clinical features	1) 871 patients from ABIDE [83]. 2) 675 subjects from ANDI [37]. (Public)	Classification of ASD and health control. Prediction of conversion to AD.	Graph Operation	Nodes features were composed of image features, while the edge weights were calculated by images and non-image features.	[AUC] Image + sex + APOE4: 0.89, image + sex + APOE4 + age: 0.85 (ADNI dataset)	[AUC] Proposed: 0.89, GCN: 0.85, MLP (Concatenation): 0.74 (ADNI dataset)
28	Chen *et al* [26]	H&E images, genomic data	1) - 4) 437, 1,022, 1,011, 515 and 538 patients from TCGA-BLCA, TCGA-BRCA, TCGA-GBMLGG, TCGA-LUAD and TCGA-UCEC respectively [23] (Public)	Survival prediction of five kinds of tumors.	Operation Attention	Co-attention mapping between WSIs and genomic features.	[C-Index] Gene: 0.527, pathology images: 0.614, all: 0.653 (overall prediction of five tumors)	[C-index] Proposed: 0.653, concatenation: 0.634, bilinear pooling: 0.621. (Overall prediction of five tumors)
29	Zhou *et al* [84]	PET images, MRI images, genomic data (SNP)	805 patients from ADNI [37] (360 with complete multimodalities). (Public)	Classification of AD and its prodromal status	Operation	Learned features of every two modalities and all three modalities were concatenated at the 1^st^ and 2^nd^ fusion stage separately.	[Accuracy] MRI + PET + SNP > MRI + PET > MRI > MRI + SNP > PET + SNP > PET > SNP (Four-class classification)	[Accuracy] Proposed > MKL [85] > SAE [86] (Direct concatenation of learned unimodal features)
30	Huang *et al* [87]	CT images, clinical features, and lab test results	1,837 studies from 1,794 patients	Classification of the presence pulmonary embolism	Operation	Compared seven kinds of fusion, including early, intermediate and late fusion. Late elastic fusion performed the best.	[AUC] Images: 0.791, clinical and lab test: 0.911, all: 0.947.	[AUC] Early fusion: 0.899, late fusion: 0.947, joint fusion: 0.893.
31	Lu *et al* [69]	Pathology images, genomics data	736 patients from TCGA-GBM/LGG [23]. (Public)	Survival prediction and grade classification of glioma tumors.	Operation Attention	Proposed a multimodal transformer encoder for co-attention fusion.	[C-index] Images: 0.7385, gene: 0.7979, images + gene: 0.8266 (Same trend for the classification task)	[C-index] Proposed: 0.8266 pathomic fusion [67]: 0.7994
32	Cai *et al* [51]	Camera/dermatoscopic images, clinical features	1) 10,015 cases from ISIC [43]. (Public) 2) 760 cases from a private dataset.	Classification of skin wounds	Operation Attention	Two multi-head cross attention to interactively fuse information from images and metadata.	[AUC] Images: 0.944 clinical features: 0.964 images + clinical: 0.974 (Private dataset)	[AUC] Poposed: 0.974, metaBlock [88]: 0.968, concatenation: 0.964 (Private dataset)
33	Jacenkow *et al* [72]	X-ray images, free-text reports	210,538 cases from MIMIC-CXR [89]. (Public)	Classification chest diseases	Attention	Finetuned unimodally pre-trained BERT models by a multimodal task.	[ACC] Images: 86.0 text: 85.1 images + text: 87.7	[ACC] Proposed: 87.7, attentive [90]: 86.8
34	Li *et al* [58]	X-ray images, free-text reports	1) 222,713 cases from MIMIC-CXR [89], 2) 3,684 cases from OpenI [81]. (Public)	Classification of chest diseases	Attention	Used different pre-trained visual-text transformer.	[AUC] Text: 0.974, image + text: 0.987 (MIMIC-CXR)	[AUC] VisualBERT [91, 92]: 0.987, LXMERT [93]: 0.984, UNITER [94]: 0.985, PixelBERT [95]: 0.953 (MIMIC-CXR)

## Data Availability

No new data were created or analyzed in this study.
